# Intravenous Iron Administration and Hypophosphatemia in Clinical Practice

**DOI:** 10.1155/2015/468675

**Published:** 2015-04-27

**Authors:** S. Hardy, X. Vandemergel

**Affiliations:** Department of General Internal Medicine, Centres Hospitaliers Jolimont, 1400 Nivelles, Belgium

## Abstract

*Introduction*. Parenteral iron formulations are frequently used to correct iron deficiency anemia (IDA) and iron deficiency (ID). Intravenous formulation efficacy on ferritin and hemoglobin level improvement is greater than that of oral formulations while they are associated with lower gastrointestinal side effects. Ferric carboxymaltose- (FCM-) related hypophosphatemia is frequent and appears without clinical significance. The aim of this study was to assess the prevalence, duration, and potential consequences of hypophosphatemia after iron injection. *Patients and Methods*. The medical records of all patients who underwent parenteral iron injection between 2012 and 2014 were retrospectively reviewed. Pre- and postinjection hemoglobin, ferritin, plasma phosphate, creatinine, and vitamin D levels were assessed. Patients who developed moderate (range: 0.32–0.80 mmol/L) or severe (<0.32 mmol/L) hypophosphatemia were questioned for symptoms. *Results*. During the study period, 234 patients received iron preparations but 104 were excluded because of missing data. Among the 130 patients included, 52 received iron sucrose (FS) and 78 FCM formulations. Among FS-treated patients, 22% developed hypophosphatemia versus 51% of FCM-treated patients, including 13% who developed profound hypophosphatemia. Hypophosphatemia severity correlated with the dose of FCM (*p* = 0.04) but not with the initial ferritin, hemoglobin, or vitamin D level. Mean hypophosphatemia duration was 6 months. No immediate clinical consequence was found except for persistent fatigue despite anemia correction in some patients. *Conclusions*. Hypophosphatemia is frequent after parenteral FCM injection and may have clinical consequences, including persistent fatigue. Further studies of chronic hypophosphatemia long-term consequences, especially bone assessments, are needed.

## 1. Introduction

In the United States, iron-deficiency anemia (IDA) affects 1-2% of adults while iron deficiency without anemia (ID) is more prevalent, affecting 11% of childbearing age women, and it is estimated that 7.8 million women have iron deficiency [1]. Symptoms of IDA usually include weakness, headache, irritability, fatigue, exercise intolerance and reduced exercise capacity [2], pagophagia, and restless legs syndrome [3]. They are often not specific and no clear clinical correlation has been shown between the degree of anemia and clinical manifestations. However, it has previously been shown that iron supplementation should be discussed in women with unexplained fatigue whose ferritin levels are less than 50 *μ*g/L, even in the absence of anemia [4]. Parenteral iron treatments are increasingly used because of their higher efficacy and fewer gastrointestinal side effects compared to oral iron therapy [5]. In Europe, ferric carboxymaltose (FCM) and iron sucrose (FS) are frequently used in this context.

Phosphate homeostasis is maintained via the bone-kidney endocrine axis, which is mainly regulated by the parathyroid hormone (PTH), vitamin D, and a group of hormones called phosphatonins, including fibroblast growth factor 23 (FGF23) [6]. In 2008 [7], the FDA has reported that 2.1% of patients treated with FCM injection developed hypophosphatemia but profound hypophosphatemia was rare. However, we have recently reported the case of a patient with profound and sustained hypophosphatemia due to phosphate diabetes [8] after FCM administration, a complication which has also been reported by others [9–11], and some patients, including those with history of bariatric surgery, are more likely to develop hypophosphatemia. Phosphate plays an important role in the organism and hypophosphatemia, especially when severe, is associated with various complications including fatigue [12], seizures [13], osmotic demyelination syndrome [14], myocardial depression [15], ventricular tachycardia [16], proximal myopathy [17], rhabdomyolysis [18], and hemolytic anemia [19]. The aim of this study was to retrospectively assess the impact of intravenous iron formulations on the phosphate level in clinical practice.

## 2. Patients and Methods

The medical records of all patients treated with parenteral iron formulation (either FCM or FS) in the Department of General Internal Medicine, Centres Hospitaliers Jolimont, Belgium (800 beds), between January 2012 and December 2014 were retrospectively reviewed. Indication, type, and dose of iron formulations were studied. Biological parameters including hemoglobin, ferritin, plasma phosphate, creatinine, PTH, and vitamin D levels were assessed before and after iron injection. Hypophosphatemia was considered moderate when the phosphate level ranged between 0.32 and 0.80 mmol/L and severe when the level was less than 0.32 mmol/L.

### 2.1. Statistical Analysis

Results are expressed as mean ± standard deviation. Comparisons were done using a *t*-test. *p* < 0.05 was considered statistically significant. Correlations were assessed using a Spearman test.

## 3. Results

During the study period, 234 patients received iron formulations. One hundred and four cases were excluded due to the absence of phosphate assessment before or after iron injection. Finally, 130 patients were included: 52 were treated with FS injection (17 men, 35 women) and 78 with FCM injection (24 men, 54 women).

### 3.1. Iron Sucrose

Among the 52 FS-treated patients, the mean dose of FS was 701 mg (quartile 200–800). Patient mean age was 55 ± 21 years. Iron was prescribed by hematologists (5 patients), geriatricians [2], gastroenterologists [4], nephrologists [1], oncologists [4], internists [3], pediatricians [2], and gynecologists [1]. Indications for FS injection were IDA in 40 cases and ID in 6 cases and 3 patients had no anemia and no ID. Moreover, 3 patients received FS injection while their hemoglobin level and iron status were not assessed. Patient characteristics are presented in Table 1. Forty patients were asymptomatic, 12 presented with fatigue, and 3 complained of dyspnea. One patient had anemia-related vertigo. The mean hemoglobin level before injection was 10.1 ± 1.99 gr/dL and it increased to 11.5 ± 2.3 gr/dL after iron administration (*p* = 0.02). The mean ferritin level rose from 64 *μ*g/L (quartile: 6–61 *μ*g/L) to 133 *μ*g/L (34–139 *μ*g/L) (*p* = 0.08). No difference in ferritin level or in hemoglobin level was noted between patients with and without fatigue (*p* = 0.63 and *p* = 0.6 for the ferritin and hemoglobin level, resp.). Before intravenous injection, 42% of patients had taken oral iron formulation which was ineffective and led to digestive intolerance in 54% and 31% of cases, respectively. The initial phosphate level measured was 1.08 ± 0.23 mmol/L and it did not change significantly following FS administration (1.00 ± 0.29 mmol/L; *p* = 0.37). Before injection, 4 patients had moderate hypophosphatemia and none had severe hypophosphatemia. After injection, 22% of patients developed hypophosphatemia with a phosphate level less than 0.80 mmol/L (they were all within normal range before injection). The lowest value after administration was 0.44 mmol/L. Hypophosphatemia duration varied between 2 and 18 weeks. However, the long-term phosphate level was not systematically assessed. No clinical manifestations of hypophosphatemia were found in the medical records. After injection, the phosphate level did not correlate with the initial hemoglobin level (rs = 0.153, *p* = 0.5), ferritin level (rs = 0.05, *p* = 0.82), or cumulated injected iron doses (rs = 0.2, *p* = 0.37).

### 3.2. Iron Carboxymaltose

Among the 78 FCM-treated patients, the mean dose of FCM was 2123 mg (quartile: 1000–2000 mg). Various physicians prescribed the drug, including haematologists (6 cases), gastroenterologists [6], oncologists [2], general internists [2], nephrologists [1], pneumologists [1], endocrinologists [1], geriatricians [1], surgeons [1], and emergency physicians [1]. Ninety percent of patients had IDA, 4% had ID, and 6% had history of IDA but did not have anemia or ID at the time of the injection. Before injection, 52% of patients had taken oral iron which was associated with gastrointestinal side effects in 20% of cases.

The initial hemoglobin level was 9.6 ± 1.6 gr/dL and it increased to 11.5 ± 1.7 gr/dL after injection (*p* < 0.001). The ferritin level was also significantly higher after injection (from 63 *μ*g/L (8–55) to 408 *μ*g/L (128–536)). No difference in hemoglobin level was observed between patients with and without symptoms of fatigue (9.6 ± 1.8 gr/dL versus 9.7 ± 1.7 gr/dL, resp.).

The initial mean phosphate level was 1.08 ± 0.18 mmol/L and it decreased to 0.82 ± 0.29 mmol/L following iron administration (*p* < 0.0001). After injection, 13% of patients had a phosphate level <0.32 mmol/L and 51% had a phosphate level <0.80 mmol/L. No difference in hypophosphatemia severity was noted between patients with history of bariatric surgery and the other patients.

Patients with severe hypophosphatemia were questioned about their symptoms, including 62% by phone interview. Among them, 55% had fatigue improvement and 30% complained of fatigue worsening while the remaining patients had no change in fatigue. In the group with fatigue worsening, the mean phosphate level was 0.55 mmol/L (0.32–0.76 mmol/L). The mean hypophosphatemia duration was 6 months (2–9 months), a long period of time which was due to the fact that patients often received other injections during the follow-up. Some patients never reached a normal phosphate level during the entire study period (2 years). The comparison of patients with and without hypophosphatemia is presented in Table 2.

## 4. Discussion

Iron is an essential nutrient for the optimal functioning of the human body. It plays a central role in hemoglobin synthesis and in many cellular processes, including oxygen transport and storage, generation of energy through oxidative phosphorylation, and enzyme activity affecting the intracellular metabolism [20]. IDA is commonly found in clinical practice, affecting 1-2% of adults in the United States [1] and accounting worldwide for 65.5 million years lived with disability [21]. Classical symptoms of IDA reported in the literature include fatigue, headache, dyspnea, and sleep disturbance secondary to restless legs syndrome [2, 22]. IDA is also known to negatively impact the physical quality of life and cognitive functions [23, 24].

However, the link between anemia and symptoms, for example, fatigue, has been poorly investigated in the literature. First, it is well known that patients may have acute or chronic profound anemia in the absence of symptoms [25, 26]. The study by Wood and Elwood did not find a correlation between the hemoglobin level and the symptoms of anemia, including fatigue, dyspnea, and palpitations [27]. Most of the trials assessing the efficacy of intravenous iron formulations have shown a rapid increase in ferritin and hemoglobin levels, assessed as primary endpoints. Some of them have also assessed clinical endpoints. A sponsored, randomized, placebo-controlled, single-blind study [28] has assessed the efficacy and safety of single-dose intravenous FCM on fatigue reduction in iron-deficient, premenopausal women with symptomatic, unexplained fatigue and normal or borderline hemoglobin level (≥115 g/L). They have shown that a single infusion of FCM improved the fatigue, mental quality of life, cognitive function, and erythropoiesis in these patients. In their study, 28% of related adverse events were observed in the treatment group versus 3.4% in the placebo group (*p* < 0.0004), including fever, urticaria, headache, and nausea. Herfs et al. [29] have shown an improvement in some clinical symptoms with FCM, including fatigue, lack of concentration, hair loss, dyspnea, and sleep disturbance in women with IDA and ID. Froessler et al. [30] have also shown an improved quality of life related to FCM injections in pregnant women with anemia but they did not assess fatigue with a validated scale and they reported 20% of side effects. In patients with inflammatory bowel disease, Evstatiev et al. [31] have shown that the quality of life remained unchanged after FCM administration in nonanemic patients with low serum ferritin level. In heavy uterine bleeding, van Wyck et al. [32] have compared FCM to oral ferrous sulfate in 325 women with postpartum IDA with hemoglobin levels less than 10 gr/L. They have shown that both treatment groups presented similar improvements in terms of quality of life scores. Another study by the same group [33] has also shown an improved quality of life in acute anemic patients. Finally, improved quality of life, functional capacity, or six-minute walk test has been reported in patients with chronic heart failure [34, 35].

In terms of efficacy, our study was similar to prospective studies showing a rapid elevation in hemoglobin and ferritin levels [36, 37]. Regarding side effects, in clinical trials, serious anaphylactic/anaphylactoid reactions have been reported in 0.1% of patients treated with FCM [38]. Other serious reactions potentially related to hypersensitivity, including pruritus, rash, urticaria, wheezing, or hypotension, were experienced by 1.5% of cases versus 3.8% of patients with hypertension [39]. Hypophosphatemia is not systematically assessed in clinical trials but when done its rate is about 2.1% [38], while other studies have reported higher rates. In the study assessing the effect of a single dose of FCM in fatigued women, Favrat et al. [28] have indeed found that 86% of patients had a phosphate level <0.8 mmol/L at day 7 which resolved spontaneously before the end of the study in almost all FCM-treated patients; however, no data on the risk of profound hypophosphatemia (<0.32 mmol/L), fatigue persistence, and hypophosphatemia development were reported. Iron-induced hypophosphatemia could play a role given that the results of some cognitive function tests do not improve after treatment. In patients with chronic kidney disease (CKD) and IDA, Macdougall et al. [40] have also found that the mean serum phosphate level decreased by 0.18 mmol/L at week 4 from baseline value but returned to normal value at week 52.

In 2010, Mani et al. [10] has reported a case of profound hypophosphatemia after FCM injection in a stable renal transplant recipient. Some case reports have been published since then [9–11] and we have recently published the case of a patient with severe hypophosphatemia due to phosphate diabetes secondary to FCM injection [8]. In our study, 22% of patients developed hypophosphatemia after FS treatment but none of them developed severe hypophosphatemia. This frequency is high but hypophosphatemia was always moderate. We found only two case reports of hypophosphatemia after iron sucrose injection [41, 42].

Phosphate plays a key role in various biological processes. In recent years, new insights into the regulation of the phosphate metabolism have been obtained, including growing evidence suggesting that factors other than the PTH and vitamin D are involved in maintaining the phosphate balance. A new class of phosphate-regulating factors, the so-called “phosphatonins,” has also been shown to play a role in phosphate-wasting diseases [43]. Among them, FGF23 is involved in various diseases, including autosomal dominant hypophosphatemic rickets/osteomalacia (ADHR) or tumor-induced osteomalacia [44]. True hypophosphatemia can be induced by a decrease in intestinal absorption, increase in urinary phosphate excretion, or acute movement of extracellular phosphate into the cells. Then, the normal renal response to phosphate depletion is to increase phosphate reabsorption, leading to the virtual abolition of urinary phosphate excretion. Most of the filtered phosphate is reabsorbed in the proximal tubule by the sodium-phosphate cotransporter in the luminal membrane [45]. A phosphate plasma level <1 mg/dL (0.32 mmol/L) can be deleterious because it can lead to cardiac arrest and severe hypophosphatemia can lead to metabolic encephalopathy and could therefore contribute to the development of central and extrapontine myelinolysis [13, 14, 16].

In this study, 13% of patients developed severe and prolonged hypophosphatemia after FCM injection. We did not find any risk factor for the development of severe hypophosphatemia and we showed that a vitamin D level initially low did not correlate with the phosphate level following injection. The mechanism underlying the development of hypophosphatemia is not known but many arguments suggest a role of FGF23. Per se, the FGF23 level is increased in ID [46, 47] but, in our retrospective study, no correlation was found between the initial ferritin level and the postinjection hypophosphatemia severity. In a prospective trial, Schouten et al. have shown that intravenous iron polymaltose injection is followed by a rapid increase in FGF23 level and a drop in phosphate and 1,25(OH)2D tubular reabsorption that persisted 3 weeks after injection. The increased FGF23 level found in their study was very high and similar to that seen in ADHR [48]. We found no difference between patients developing hypophosphatemia and nonhypophosphatemic patients except for the iron cumulative dose. To the best of our knowledge, there are no published studies conducted in patients without CKD in which the impact of FCM injection on phosphate metabolism has been assessed. However, in a post hoc study, Prats et al. [49] have assessed the effect of FCM injection on the serum phosphate and c-terminal FGF23 levels in nondialysis CKD patients and no difference between hypophosphatemic and nonhypophosphatemic patients was found in terms of initial 25(OH)D, ferritin, and hemoglobin levels.

Our study has some limitations, in particular its retrospective design and the fact that the value of the FGF23 level was not recorded. However, if hypophosphatemia is secondary to the increase in FGF23 as demonstrated in other studies, hypophosphatemia duration, several months in some cases, raises questions about the long-term side effects of the drug on bone.

Indeed, recent reports suggest that FGF23 may have a phosphate-independent effect on bones [50] with suppression of the osteoblast differentiation and matrix mineralization in a fetal rat calvaria cell line overexpressing FGF23. These consequences have been validated by two case reports of osteomalacia induced by intravenous iron sucrose administration [51, 52]. It is surprising because iron sucrose is less often complicated by hypophosphatemia. Therefore, patients with history of bariatric surgery need particular attention. They represented 18% of patients treated with FCM in our study. It is well known that metabolic bone disease is a concern during the follow-up of these patients [53] and an evolving concept of bariatric osteomalacia has emerged recently [54]. In our study, a history of bariatric surgery was not a risk factor for developing hypophosphatemia, but 60% of patients with history of bariatric surgery developed hypophosphatemia which, over the long term, could contribute to bone-specific complications. Moreover, 30% of our patients with FCM-induced hypophosphatemia complained about fatigue worsening. It is not known, however, if correcting hypophosphatemia could improve their symptomatology. At least, practitioners should keep in mind that it is indicated for ID. Indeed, in our study 3.8% of patients received iron formulations without hemoglobin or ferritin level evaluation. Blood test should be performed before injection and the phosphate level assessed both before and after injections.

Parenteral iron formulations are increasingly prescribed but their long-term side effects remain unknown, in particular the long-term bone impact of hypophosphatemia (13% in our study). In cystic fibrosis patients, respiratory deterioration has been reported after treatment [55] and intravenous iron seems to have deleterious effects on mononuclear cells, oxidative stress, and apoptosis [56].

In conclusion, we found that parenteral FCM injection is often followed by hypophosphatemia which may be profound and last a long time without immediate clinical consequences except fatigue in some cases. Studies evaluating oral phosphate supplementation and its long-term consequences are needed because the biological change in phosphate homeostasis after injection is similar to that observed in diseases such as tumor-induced osteomalacia [57] or X-linked hypophosphatemic rickets [58].

## Figures and Tables

**Table 1:** Patient characteristics.

	Iron sucrose	Iron carboxymaltose
*N*	52	78
Men/women	17/35	24/54
Active cancer	7	16
Chronic kidney disease	7	10
History of bariatric surgery	5	15
Inflammatory bowel disease	5	4
Gynecologic losses	3	7
Anemia	40	70
Iron deficiency without anemia	6	3
Fatigue	23%	29%
Asymptomatic	76%	67%
Phosphate level <0.81 mmol/L	22%	51%
Phosphate level <0.32 mmol/L	0	13%

**Table 2:** Comparison between patients developing hypophosphatemia after FCM injection and patients without hypophosphatemia.

	Patients with hypophosphatemia	Patients without hypophosphatemia	
*N*	38	40	
Women/men	27/11	27/13	NS
Age	57 ± 11	58 ± 19	NS
Vitamin D level before injection (ng/mL)	21.9 ± 16.9	20 ± 10	NS
Hemoglobin (g/L)	9.5 ± 1.3	9.7 ± 1.8	NS
Ferritin (*µ*g/L)	44 (6.25–55)	80 (9–95)	*p* = 0.14
Cumulated dose (mg)	2092 ± 2002	1350 ± 860	*p* = 0.04
